# The Citizen Doctor1Read at the meeting of the eighth district branch of the Medical Society of the State of New York held at Batavia September, 25, 1908.

**Published:** 1909-04

**Authors:** Edward Munson

**Affiliations:** Medina, N. Y.


### The Citizen Doctor[1]

BY EDWARD MUNSON, M. D., Medina, N. Y.

UPON an occasion such as this, when physicians get together to exchange views and invite discussion upon subjects medical, one ought, perhaps, to preface with an apology, a paper which, ignoring science and technic, seeks but to deal briefly with certain broad conditions of interest and importance to us as medical men.

Modern life is so complex a thing, and the medical science of today so exigent, that we are sorely tempted in our pursuit of the latter to let the other completely escape us. Too often does the busy doctor, divorcing himself completely from civic affairs, emulate the cynic's part, and exclaim with Tennyson's pessimist, "I will bury myself in my books and the Devil may pipe to his own." We are citizens before we are doctors, and other things being equal, I hold him to be the best doctor who is at the same time the best citizen.

Our profession has ever been regarded as an honorable one, and justly so. It brings us into the very closest contact with our fellow men. Into our ears are breathed such secrets as only the confessional may know. Before our eyes are lifted masks which have hidden horrors no priest has ever guessed. We are bidden to share in the simple pleasures of the poor: to revel in the palaces of the rich. We lift the new born infant up to receive its mother's first greeting, and we sit by the bedside of the nearly-departed, while "pallid death upon the black horse" keeps like vigil with us just outside the door. Surely, than the physician no one can better echo the sentiment of the old Roman, "I am a man, and nothing which concerns mankind can be an alien subject to me."

Now, to begin with, it seems to me that medical men should take a keener interest in politics. Of course, I do not mean that they should become "politicians" in the vernacular sense of the term, but that they should keep well abreast of national, state and

---

[1] Read at the meeting of the eighth district branch of the Medical Society of the State of New York held at Batavia September, 25, 1908.

local political matters; that they should attend primaries when possible and lend their influence to the best men and the best measures, and that, when occasion calls, they should rise above the partisan, and contribute their mite to uplift the plane of our common citizenship. Then, too, the citizen doctor should be ever mindful of the importance of the matter of education, as it is conducted in our public school system. There is much in this that will appeal to him in his dual capacity as citizen and physician. An official connection with school boards extending over a period of twenty years has opened my eyes to some serious defects in a system, which at the same time possesses many excellencies.

One may note with pleasure a general improvement in matters of ventilation, the heating and the lighting of schools. There has been advancement made in the direction of sanitary closets, in better constructed seats, in text books, libraries and apparatus for laboratory work. There has been a steady and remarkable increase in the qualifications required for teaching, and there has recently been a belated and somewhat dilettante examination for bodily defects in the pupils. But, when all of these things have been conceded, the fact remains that, as regards educational matters, there is still something rotten in the state of New York. One may well doubt whether, in the fundamentals, the youth of today are as well grounded as were those of thirty years ago. There are too many subjects crowded into a curriculum of eight years. The pupil, forsooth, must needs have a smattering of physiology, of music, of literature and of art. He has "brushed with extreme flounce the circle of the sciences," but the three R's have suffered in the process."

Already there are signs of a reaction against these methods. Col. Charles W. Larned, reporting the results of this year's examinations for entrance at the West Point Military Academy, where there were twenty failures out of thirty-seven applicants from New York State alone, remarks that "this is surely a state of affairs that should make the judicious grieve and our educators sit up and take notice." In the city of Rochester, only the other day, there was held a meeting of citizens which entered a vigorous protest against the cramming fad. More power to their elbows! to which I invite my medical confreres to contribute. Again the physician should be ever in the van of those who labor to forward sanitation and the prevention of disease in the state. He should by voice and vote hold up the hands of the health-boards in their efforts to minimise the spread of infection and contagion, and no one is so well qualified as he to combat the ignorance and superstition which obstruct such measures

Finally, if he would fulfill his highest duties as citizen doctor, he should lift up his voice, in season and out of season, against the three mighty vices, which have always afflicted and which still afflict his fellowmen: to-wit, the social evil, race suicide and intemperance. Let us ceaselessly hammer into the minds of our youth that "the gods are just and of our pleasant vices make instruments to scourge us."

He who sees as does the physician, the insidious, but merciless, ravages of the gonococcus and the microbe of syphilis, the countless thousands of innocent women who invite pus tubes unaware, the bright young victims of paresis syphilitica, and the maniacal miseries of delirium tremens, needs no dogma of hell-fire to point his text. Better than the minister, more forcefully even than the priest, can the wise and trusted medical counselor point out with warning finger the pitfalls which beset the path of self-indulgent youth. Nor should he be disheartened because of a world around him teeming with misery and vice. For though often times he may find himself in agreement with Huxley, when he says, "I know no study which is so unutterably saddening as that of the evolution of humanity as it is set forth in the annals of history," yet he must needs agree also with Huxley's conclusion that "one should rejoice in the good man, forgive the bad man and pity and help all men to the best of one's ability."

On the subject of criminal abortion but little need be said to an audience such as this. The vast majority of medical men stand four square in this matter, while to the criminals within our ranks, who rate the value of a fetal life at the best price which they can exact from their guilty partners in crime, appeal is useless. Hasten the day when they will be more and more regarded by the profession as "mere Pariahs of the outer world," and relegated in ever increasing numbers to the prison cells, which the law and the state hold ready for them.

And, now, having attempted thus briefly and, I fear, somewhat crudely, to outline a few of the directions where the duties of the physician merge into those of the citizen, I desire, in conclusion, to speak of one quality which, fostered and developed as it may be, will prove of the highest value to us, both as citizens and as doctors. Aside from purely scientific and technical attainments, I know of no asset so valuable to the physician as a healthy optimism. On the other hand, my imagination fails to depict a figure more cheerless and depressing than that of the medical pessimist. He would outdo Pandora, and from the all-but-emptied box of blessings would let even Hope escape. Surely, the cheering smile and the hopeful word should occupy no mean space in the medical armamentarium.

Now, to some, the attainment of a true optimism may be no easy task. One's outlook upon life has its roots deep in one's philosophy of life. Whatsoever a man believes, that he is. To my thinking two prerequisites are needful: belief in a supreme being and faith in a continued existence. Our profession has been often charged with a leaning toward materialism. If ever true, I believe that charge will not hold good today. Impatient of dogma it may have been; intolerant of creeds which have waged a losing fight against the progress of all science. But if believing in a future life, one can add only so much of faith as glimmers through the shell of that great agnostic, Herbert Spencer, when he speaks of "an infinite and eternal energy from which all things proceed," or of the unorthodox Mathew Arnold, when he defines deity as "the power not ourselves, which makes for righteousness," then one may be considered to have made a good start towards looking on the bright side of life. And the true physician will be content, if, when his life's fight against disease and death is finished, he can be written down, in the beautiful words of Robert Browning, the poet optimist, as

"One who never turned his back but marched breast forward,
Never doubted clouds would break,
Never dreamed though right were worsted wrong would triumph,
Held we fall to rise, are baffled to fight better, sleep to wake."

